# Continuing professional development needs in pain management for Canadian health care professionals: A cross sectional survey

**DOI:** 10.1080/24740527.2022.2150156

**Published:** 2023-01-23

**Authors:** Craig M. Dale, Iacopo Cioffi, Christine B. Novak, Franklin Gorospe, Laura Murphy, Deepika Chugh, Judy Watt-Watson, Bonnie Stevens

**Affiliations:** a Lawrence S. Bloomberg Faculty of Nursing, University of Toronto, Toronto, Canada; b Tory Trauma Program, Sunnybrook Health Sciences Centre, Toronto, Canada; c Faculty of Dentistry, University of Toronto, Toronto, Canada; d Department of Surgery, The Hospital for Sick Children, Toronto, Canada; e Toronto General Hospital, University Health Network, Toronto, Canada; f Department of Pharmacy, University Health Network, Toronto, Canada; g Toronto Rehabilitation Institute, University Health Network, Toronto, Canada; h Department of Dentistry, Mount Sinai Hospital, Toronto, Canada; i Research Institute, The Hospital for Sick Children (SickKids), Toronto, Canada

**Keywords:** pain education, postlicensure clinician, continuing professional development, competency-based education, survey

## Abstract

**Background:**

Continuing professional development is an important means of improving access to effective patient care. Although pain content has increased significantly in prelicensure programs, little is known about how postlicensure health professionals advance or maintain competence in pain management.

**Aims:**

The aim of this study was to investigate Canadian health professionals’ continuing professional development needs, activities, and preferred modalities for pain management.

**Methods:**

This study employed a cross-sectional self-report web survey.

**Results:**

The survey response rate was 57% (230/400). Respondents were primarily nurses (48%), university educated (95%), employed in academic hospital settings (62%), and had ≥11 years postlicensure experience (70%). Most patients (>50%) cared for in an average week presented with pain. Compared to those working in nonacademic settings, clinicians in academic settings reported significantly higher acute pain assessment competence (mean 7.8/10 versus 6.9/10; *P* < 0.002) and greater access to pain specialist consultants (73% versus 29%; *P* < 0.0001). Chronic pain assessment competence was not different between groups. Top learning needs included neuropathic pain, musculoskeletal pain, and chronic pain. Recently completed and preferred learning modalities respectively were informal and work-based: reading journal articles (56%, 54%), online independent learning (44%, 53%), and attending hospital rounds (43%, 42%); 17% had not completed any pain learning activities in the past 12 months. Respondents employed in nonacademic settings and nonphysicians were more likely to use pocket cards, mobile apps, and e-mail summaries to improve pain management.

**Conclusions:**

Canadian postlicensure health professionals require greater access to and participation in interactive and multimodal methods of continuing professional development to facilitate competency in evidence-based pain management.

## RÉSUMÉ

**Contexte**: Le développement professionnel continu est un moyen important d’améliorer l’accès à des soins efficaces pour les patients. Bien que le contenu lié à la douleur ait augmenté de manière significative dans les programmes préalables à l’autorisation d’exercer, on sait peu de choses sur la façon dont les professionnels de la santé après licenciés améliorent ou maintiennent leurs compétences en matière de gestion de la douleur.

**Objectifs**: Étudier les besoins, les activités et les préférences de développement professionnel continu des cliniciens canadiens en matière de gestion de la douleur.

**Méthodes**: Enquête Web transversale d’auto-évaluation.

**Résultats**: Le taux de réponse au sondage était de 57 % (230/400). Les répondants étaient principalement des infirmières (48 %), des diplômés universitaires (95 %), des employés en milieu hospitalier universitaire (62 %), avec ≥11 ans d’expérience après l’obtention du permis (70 %). La plupart des patients (> 50 %) pris en charge dans une semaine moyenne présentaient des douleurs. Comparativement à ceux qui travaillent dans des milieux non universitaires, les cliniciens en milieu universitaire ont signalé une compétence d’évaluation de la douleur aiguë significativement plus élevée (moyenne de 7,8/10 contre 6,9/10; P < 0,002) et un meilleur accès aux consultants en gestion de la douleur (73 % contre 29 %; P < 0,0001). La compétence d’évaluation de la douleur chronique n’était pas différente entre les groupes. Les principaux besoins d’apprentissage comprenaient la douleur neuropathique, la douleur musculo-squelettique et la douleur chronique. Les modalités d’apprentissage récemment achevées et préférées étaient respectivement informelles et basées sur le travail: lecture d’articles de journaux (56 %, 54 %), apprentissage indépendant en ligne (44 %, 53 %) et participation à des visites à l’hôpital (43 %, 42 %); 17 % n’avaient effectué aucune activité d’apprentissage de la douleur au cours des 12 derniers mois. Les répondants hors les médecins et ceux employés dans des milieux non universitaires étaient plus susceptibles d’utiliser des cartes de poche, des applications mobiles et des résumés par e-mail pour améliorer la gestion de la douleur.

**Conclusions**: Les professionnels de la santé canadiens après l’obtention du permis d’exercice ont besoin d’un meilleur accès et d’une plus grande participation aux méthodes interactives et multimodales de développement professionnel continu pour faciliter la compétence en gestion de la douleur fondée sur des données probantes.

## Introduction

Inadequate knowledge and skills among practicing health care professionals (HCPs) are persistent barriers to effective pain management and positive patient outcomes.^1^ Due to the growing prevalence and societal burden of pain, continuing professional development (CPD) has emerged as an important means of improving access to evidence-based pain care.^2^ However, some HCPs continue to express discomfort in addressing pain concerns due to unmet learning needs, which may contribute to poor outcomes.^3^ Despite efforts to increase knowledge of pain prevention and management among prelicensure HCPs, there is scant understanding of postlicensure initiatives to advance and/or maintain evidence-based pain practice.^4^ Understanding postlicensure HCPs’ pain education needs, activities, and preferred learning modalities is an important starting point for the development of effective competency based CPD pain resources.

Internationally, millions of individuals suffer from pain each year as a result of disease, trauma, and surgery.^5^ Although pain is among the most common reasons patients seek medical attention, the management of pain remains inadequate across settings, with a substantial proportion of patients reporting unrelieved pain.^6^ Up to 75% of patients report moderate to severe acute pain^7^ and up to 60% of patients experience chronic pain following common surgical procedures.^8^ Some individuals seek the help of more than one HCP for chronic pain diagnosis and treatment. However, other individuals may choose not to seek medical assistance due to a perceived lack of provider expertise or empathy.^9^ Poorly managed chronic pain carries both human and economic costs for individuals, families, and society because it undermines patients’ ability to participate in relationships, education, and employment. Chronic pain is also more frequently experienced in populations affected by social and economic inequities, which may exacerbate health disparities and the incremental costs to health systems.^10,11^

CPD is an umbrella term for a range of activities undertaken by HCPs to acquire the knowledge and skills needed to deliver high-quality care throughout their careers.^3^ CPD activities relevant to pain may include but are not limited to formal courses (e.g., classroom learning, online courses, workshops), informal activities (e.g., reading journal articles, attending professional conferences), and work-based learning (e.g., hospital rounds, case discussions).^4^ CPD has traditionally focused on passive delivery formats typified by experts providing learning content in a lecture format.^12^ More recently, CPD has progressed toward competency-based CPD, which moves beyond conceptual knowledge gain (e.g., input) toward an individual’s capacity to successfully and empathically perform a task (e.g., outcome). One example is Project ECHO (the Extension for Community Healthcare Outcomes), which uses telemedicine to connect HCPs working in rural or nonacademic settings with pain specialists, aiming to enhance their competencies in helping people to manage their chronic pain.^13^ Project ECHO is characteristic of competency-based CPD in its use of active problem solving, real-world cases, application of evidence-based guidelines, and feedback mechanisms.^3^

The International Association for the Study of Pain (IASP) has endorsed the interprofessional pain consensus competencies for prelicensure HCP training that address the IASP four pain curricula domains: multidimensional nature of pain, pain assessment and measurement, management of pain, and clinical conditions.^14^ The pain competency framework includes recommendations for active learning methods such as objective appraisal of performance and personalized learning feedback. Although developed for prelicensure HCPs, the pain competency framework has been identified as an ideal approach for thinking about knowledge gaps among licensed HCPs.^4^ Despite prior research identifying the success of continuing education in pain, HCPs report variable access to CPD resources aligned with their self-identified learning needs, preferred learning formats, and perceptions of quality.^15,16^ CPD educators, health care organizations, and accrediting bodies would benefit from further insight regarding HCP learning needs and preferences to determine how best to meet those needs.

The University of Toronto Center for the Study of Pain (UTCSP), a collaborative partnership of the Faculties of Medicine, Nursing, Dentistry, and Pharmacy at the University of Toronto, upholds a mission to create and disseminate knowledge on pain across the life span and foster clinical excellence through interdisciplinary collaboration.^17^ To meet its goal to develop educational programs in pain, the UTCSP Education Subcommittee sought to better understand Canadian postlicensure clinicians’ CPD needs with regard to pain. The specific study objectives were to describe HCPs’ self-identified pain learning needs according to the IASP competency framework, recent continuing education activities, and preferred learning modalities and explore individual HCP characteristics that may inform future CPD opportunities. We hypothesized that HCPs working in academic (i.e., university-affiliated) hospital settings would have greater access to pain knowledge resources, and therefore different self-rated pain assessment competencies and learning needs, in comparison to those working in nonacademic hospitals or other settings.

## Methods

### Overview

We conducted a self-administered, cross-sectional, deidentified web survey of licensed Canadian HCPs’ pain CPD needs, activities, and preferred learning modalities. We aimed to include diverse postlicensure HCPs employed in a hospital or outpatient setting at the time of the survey.

### Survey Development

Seven clinician-scientists generated survey items: three nurses, one physician, two dentists, and a physiotherapist. In addition to participant demographics (e.g., professional role, care setting, patient populations), survey items pertained to self-rated acute and chronic pain assessment competency, evidence-based pain appraisal and management resources, and future areas for professional development organized according to the four pain competency domains.^14^ Item reduction for the survey occurred through an iterative process among the study investigators. The survey was then sent to five pain experts who were not involved in item generation for face and content validity, discriminability, utility, and clarity.^18^ Five different clinicians participated in cognitive interviews to confirm comprehension and congruence of domains and items and to determine the 10-min time to survey completion (Supplement 1).^19^

### Sample and Methodology

We estimated a definitive sample size for this survey derived from a UTCSP-Interfaculty Pain Curriculum (IPC) member database of approximately 400 clinicians including nurses, physicians, pharmacists, dentists, and diverse allied health professionals. These members had previously registered with the UTCSP to participate in research and educational opportunities. Assuming a population of this size, a 95% confidence level, and a margin of error of 5%, it was estimated that 196 clinicians would be needed to complete the survey. Delivery and management of the survey was accomplished using SurveyMonkey, a secure web-based application designed exclusively to support data capture for survey studies.^20^ The survey items had a multiple-choice response format with the exception of two self-rating scales (0–10) for pain assessment competence, where 0 indicated *not competent* and 10 indicated *highly competent*. We followed the modified Dillman method, which aims to maximize survey response and minimize project bias.^21^ This included an introductory e-mail informing eligible respondents of the forthcoming survey, a personalized e-mail with survey link, and three reminder e-mails for nonresponse. In addition, we used the UTCSP website and Twitter account to increase awareness of the survey. There was no compensation for survey participation.

### Ethics

The study received approval from the University of Toronto Research Ethics Board (#36505). The web survey preamble outlined the purpose of the survey, data collection, and management strategies including the protection of privacy, and the anticipated time commitment to complete all items. Respondents were required to acknowledge their consent to participate to access the survey.

### Analysis

Survey data were downloaded from SurveyMonkey to Microsoft Excel (v16.63.1) for analysis. Reponses to each question were collated and analyzed by comparing groups defined by work setting (academic versus nonacademic) for professional role, highest level of education, years of professional work experience, number of patients cared for each week, self-reported pain assessment competency, access to pain specialists, and CPD activities and preferences. Categorical data were summarized as frequencies and percentages. Continuous data were summarized using mean ± standard deviations. The chi-square and/or Fisher’s exact tests were used to examine differences between groups. An independent statistician performed all analyses using SAS 9.4 (SAS Institute Inc., Cary, NC, USA). A *P* value <0.05 was considered statistically significant. To improve the transparency of reporting, we followed the Consensus-Based Checklist for Reporting of Survey Studies (Supplement 2).^22^

## Results

Over an eight-week period (December 2018 and January 2019), a total of 230 HCPs completed the survey, resulting in a 57% return rate. Respondents were nurses (registered nurses and/or nurse practitioners; 47.5%), pharmacists (16.5%), physicians (13.0%), physiotherapists (10.8%), occupational therapists (4.3%), dentists (3.9%), and other health professionals (3.0%). The majority were university educated (95%), employed in an academic hospital setting (62%), with ≥11 years (70%) postlicensure clinical experience (Table 1). Education and years of experience did not differ significantly between HPCs working in academic compared to nonacademic settings. Patient groups encountered in practice included adults (93%), older adults (80%), adolescents (36%), and infants/children (25%); 19% worked with all patient groups in their current role (Table 2). Those working in nonacademic settings more often cared for high volumes of patients (>100) in an average week compared to those in academic settings (*P* = 0.004). However, regardless of work setting, most patients (>50%) cared for in an average week reported pain, with musculoskeletal problems predominating.

**Table 1.** Respondent characteristics across academic and nonacademic settings.

Categories		Total, n (%)	Academic group	Nonacademic group	
Total		230	143	87	*P* value
Regulated health professional	Registered nurse	73 (31.7)	49	24	0.213
	Nurse practitioner	38 (15.8)	21	17	
	Pharmacist	38 (16.5)	18	19	
	Physician	30 (13.0)	24	6	
	Physiotherapist	25 (10.87)	16	9	
	Occupational therapist	10 (4.35)	5	5	
	Dentist	9 (3.91)	5	4	
	Speech-language pathology	2 (0.87)	2	0	
	Other	5 (2.17)	3	2	
Highest level of education	Baccalaureate	85 (36.9)	48	37	0.168
	Master’s degree	101 (43.9)	62	39	
	Doctorate/PhD	27 (11.7)	18	9	
	Diploma/college	11 (4.7)	10	1	
	Postdoctorate	6 (2.6)	5	1	
Years in profession	>30 Years	45 (19.5)	25	20	0.253
	21–30 Years	53 (23.0)	28	25	
	11–20 Years	64 (27.8)	43	21	
	6–10 Years	41 (17.8)	27	14	
	0–5 Years	27 (11.7)	20	7	
Clinical setting	Hospital	179 (77.8)	143	36	<0.00001*
	Ambulatory clinic/office	49 (21.3)			
	Rehabilitation	26 (11.3)			
	Pharmacy	16 (6.9)			
	Continuing care	12 (5.2)			
	Other practice setting	11 (4.7)			
	Urgent care clinic	1 (0.4)			
	Hospice	1 (0.4)			
Number of patients cared for each week	>150	6 (2.6)	2	4	0.004*
	101–150	8 (3.4)	2	6	
	51–100	50 (21.7)	25	25	
	<50	166 (72.1)	114	52	
Percentage of patients in pain each week	>50	148 (64.3)	104	44	0.001*
	41–50	18 (7.8)	13	5	
	31–40	17 (7.3)	9	8	
	21–30	21 (9.1)	6	15	
	11–20	16 (6.9)	7	9	
	0–10	10 (4.3)	4	6	
Access to acute pain specialist/team		129 (56.0)	104	25	<0.0001*
Access to chronic pain specialist/team		106 (46.0)	82	24	<0.0001*

*P < 0.05 was considered statistically significant.

**Table 2.** Patient conditions.

	Adults, *n* (%)	Older adults, *n* (%)	Adolescents, *n* (%)	Infants/children, *n* (%)
Total	214	184	83	58
Musculoskeletal	142 (66.36)	120 (56.07)	53 (63.86)	32 (55.17)
Cardiovascular	123 (57.48)	113 (52.80)	13 (15.66)	12 (20.6)
Critical illness	110 (51.4)	96 (44.86)	29 (34.94)	22 (37.93)
Neurological	107 (50)	110 (47.20)	28 (33.73)	17 (29.31)
Psychiatric	105 (49.07)	86 (40.19)	42 (50.6)	18 (31.03)
Palliative	10 3 (48.13)	96 (44.86)	10 (12.05)	6 (10.30)
Trauma	96 (44.86)	82 (38.32)	35 (42.17)	25 (43.10)
Endocrine	95 (44.93)	89 (41.59)	22 (26.51)	16 (27.59)
Oncology	95 (44.93)	84 (39.25)	18 (26.69)	13 (22.41)
Respiratory	71 (33.18)	63 (29.44)	23 (27.71)	22 (37.93)
Organ failure	67 (31.31)	57 (26.64)	13 (15.66)	9 (15.52)
Cognitive	64 (29.91)	60 (28.04)	16 (19.28)	13 (22.41)
Infectious	60 (28.04)	53 (24.77)	23 (27.71)	19 (32.76)
Substance use	60 (28.04)	45 (21.03)	20 (24.10)	6 (10.30)
Oral/craniofacial	50 (23.36)	40 (18.69)	23 (27.71)	16 (27.59)

### Pain Assessment and Treatment

**Figure 1.**
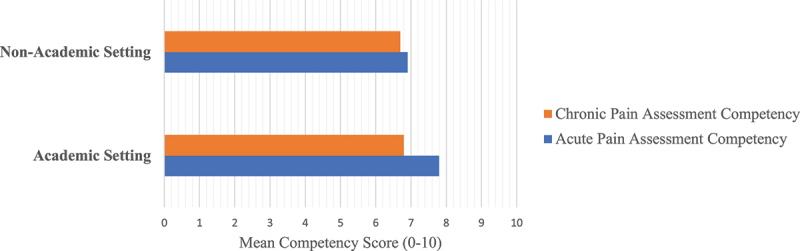
Self-reported acute and chronic pain assessment competency across academic and nonacademic settings.

Respondents employed in academic settings had significantly higher self-rated acute pain assessment competence (7.8/10 versus 6.9/10; *P* = 0.002) compared to those employed in nonacademic settings. There was no difference in chronic pain assessment competence between the group settings (Figure 1). Across respondent categories, the reported range of tools routinely used to assess patients included unidimensional self-report pain scales (31%–84%), behavioral pain scales (2%–29%), and multidimensional pain scales (15%–27%). Resources used alone or in combination to make treatment choices by all categories of respondents included patient self-report (73%), patient goal setting (61%), and professional consultation (53%); protocols (44%), guidelines (43%), and standardized clinical order sets (40%) were used less frequently (Table 3). Over half (56%) of all respondents reported having access to an acute pain specialist/team to facilitate treatment; however, slightly less than half (46%) had access to a chronic pain specialist/team. Those employed in academic hospital settings had significantly greater access to acute (73% versus 29%; *P* = 0.0001) and chronic (57% versus 27%; *P* = 0.0001) pain specialists (Table 1).

**Table 3.** Pain assessment and management resources.

	Category	Tool/resource	*n* (%)
Pain assessment tools	Unidimensional tools	Numeric rating scale	195 (84.7)
		Verbal rating scale	115 (50.0)
		Visual analog scale	93 (40.4)
		PQRST mnemonic	76 (33.0)
		Faces Pain Scale	73 (31.8)
	Multidimensional tools	Brief Pain Inventory	64 (27.8)
		McGill Pain Questionnaire	35 (15.2)
		Pain Disability Index	39 (16.9)
	Observational tools	Behavioral Pain Scale	67 (29.1)
		Critical-Care Pain Observational Tool	46 (20)
		Faces, Legs, Activity, Cry, Consolability Scale	16 (6.9)
		Premature Infant Pain Profile	5 (2.1)
	None	No tool used	5 (2.1)
Pain treatment resources	Patient	Patient self-report	168 (73.0)
		Patient goal	142 (61.7)
	Professional	Consultation	123 (53.4)
	Guideline/tool	Protocol/policy	102 (44.3)
		Expert guideline	100 (43.4)
		Clinical order set	94 (40.8)
		Online reference	65 (28.2)
	Diagnostic evaluation	Physiological testing	35 (15.2)
		Drug screen	35 (15.2)
		Quantitative sensory test	20 (8.7)
	None	No resource used	15 (6.5)

PQRST mnemonic: P = palliative or precipitating factors, Q = quality of pain, R = region or radiation of pain, S = subjective descriptions of pain severity, and T = timing/temporal nature of pain.

### Pain Competency Development Needs

**Figure 2.**
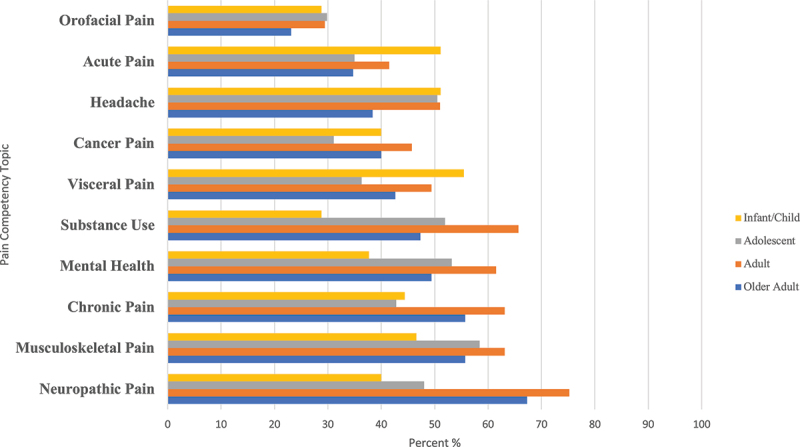
Competency development needs for populations/clinical conditions.

Self-identified pain development needs organized by the pain competency domains included (1) “multidimensional nature of pain” domain: pain physiology (59%), consequences of pain (50%), and pain theory (48%); (2) “assessment and measurement of pain” domain: pain assessment tools/scales (60%), interprofessional collaboration (57%), and tool reliability/validity (52%); (3) “management of pain” domain: psychological/cognitive methods (57%), pain management planning (54%), and pharmacological methods (54%; Table 4). For development needs related to the fourth domain, “populations/clinical conditions,” the top conditions identified were neuropathic pain for older adults (67%) and adults (75%), musculoskeletal pain (58%) for adolescents, and visceral pain (55%) for infants/children (Figure 2).

**Table 4.** Pain competency development needs.

Pain competency domain	Categories	*n* (%)
Multidimensional nature of pain	Pain physiology	137 (59.5)
	Consequences of pain	116 (50.4)
	Pain theory	111 (48.2)
	Ethical standards	95 (41.3)
	Legal issues	91 (39.5)
	Pain terminology/classifications	87 (37.8)
	Pain epidemiology	81 (35.2)
	None	43 (18.7)
Assessment and measurement of pain	Pain assessment tools/scales	138 (60.0)
	Interprofessional collaboration	133 (57.8)
	Tool reliability/validity	120 (52.1)
	History and physical exam	110 (47.8)
	Laboratory/imaging investigations	75 (32.6)
	None	33 (14.3)
Management of pain	Psychological/cognitive methods	133 (57.8)
	Pain management planning	125 (54.3)
	Pharmacological methods	125 (54.3)
	Substance use disorder	123 (53.4)
	Psychological issues	120 (50.4)
	Patient/family beliefs	116 (50.4)
	Goals of pain management	113 (49.1)
	Access to treatment	108 (46.9)
	None	30 (13.0)

### Completed CPD Activities and Preferences

In the 12-month period immediately preceding survey completion, the most frequent CPD activities related to pain were reading journal articles (56%), online independent learning (44%), and attending hospital rounds (43%); 17% had not completed any pain learning activities (Table 5). There was no significant difference in CPD preference according to academic work setting, professional role, education, or years of experience for reading journal articles or online independent learning. Participation in hospital rounds was preferred by respondents employed in academic settings (*P* = 0.007). Nonphysicians (e.g., nurses, pharmacists, dentists, and allied health professionals), when compared to physicians, had a significantly greater preference for attending formal courses (*P* = 0.02) and receiving e-mail updates summarizing the latest pain evidence (*P* = 0.01). Those with experience of ten years or less (*P* = 0.04) and those without a graduate degree (*P* = 0.03) preferred attending international and local conferences, respectively. There were no significant differences in preference across respondent categories for use of internet resources, including online courses, social media sites, or live webcasts. Across academic and nonacademic work categories, tools perceived as potentially useful for advancing evidence-based pain treatment at the point of care included mobile apps (35%–51%), pocket cards (21%–38%), e-mail updates (18%–33%), and video instruction (11%–41%); however, pocket cards and/or e-mail updates were more frequently preferred by those working in nonacademic settings (Table 6).

**Table 5.** Comparison of completed and preferred learning modalities across academic and nonacademic settings.

Variable	Completed ≤ 12 months, *n* (%)	Preferred academic group, *n* (%)	Preferred nonacademic group, *n* (%)	*P* value
Reading journal articles	129 (56.0)	77 (53.8)	47 (54.0)	0.98
Reading textbooks	40 (17.3)	27 (18.8)	17 (19.4)	0.90
Hospital rounds	101 (43.9)	70 (48.9)	27 (31.0)	0.007*
Online learning	103 (44.7)	75 (52.8)	46 (52.8)	0.950
E-mail	70 (30.4)	43 (30.0)	25 (28.7)	0.927
Local conferences	57 (24.7)	58 (40.5)	45 (51.7)	0.098
National conferences	51 (22.1)	47 (32.8)	25 (28.7)	0.559
Webcast (live)	43 (18.7)	46 (32.1)	37 (42.5)	0.112
Webcast (recorded)	36 (15.6)	47 (32.8)	36 (41.3)	0.192
Social media	34 (14.7)	21 (14.6)	12 (13.7)	0.851
International conferences	31 (13.4)	41 (28.6)	16 (18.3)	0.085
Formal courses	29 (12.6)	55 (38.4)	34 (39.0)	0.925
Journal club	28 (12.1)	30 (20.9)	12 (13.7)	0.171
Simulation	6 (2.6)	24 (16.7)	16 (18.3)	0.755
None	40 (17.3)	16 (11.1)	7 (8.0)	0.503

*P < 0.05 was considered statistically significant.

**Table 6.** Comparison of pain tool preferences across academic and nonacademic settings.

Variable	Category	Total, *n* (%)	Academic, *n* (%)	Nonacademic, *n* (%)	*P* value
Acute pain assessment	Mobile app	104 (45.2)	61 (42.6)	43 (49.4)	0.317
	Pocket card	81 (35.2)	43 (30.0)	38 (43.6)	0.036*
	Video instruction	63 (27.3)	37 (25.8)	26 (29.8)	0.508
	E-mail update	45 (19.5)	21 (14.6)	24 (27.5)	0.016*
	Social network	26 (11.3)	17 (11.89)	9 (10.3)	0.720
Chronic pain assessment	Mobile app	118 (51.3)	68 (47.5)	50 (57.4)	0.144
	Pocket card	82 (35.6)	43 (30.0)	39 (44.8)	0.023*
	Video instruction	65 (28.2)	41 (28.6)	24 (27.5)	0.859
	E-mail update	54 (23.4)	28 (19.5)	26 (29.8)	0.073
	Social network	30 (13.0)	21 (14.6)	9 (10.3)	0.343
Analgesic dosing guide	Mobile app	118 (51.3)	69 (48.2)	49 (56.3)	0.235
	Pocket card	88 (38.2)	51 (35.6)	37 (42.5)	0.298
	Video instruction	34 (14.78)	22 (15.3)	12 (13.7)	0.741
	E-mail update	49 (21.3)	26 (18.1)	23 (26.4)	0.138
	Social network	13 (5.6)	9 (6.2)	4 (4.6)	0.589
Drug interactions	Mobile app	119 (51.7)	69 (48.2)	50 (57.4)	0.174
	Pocket card	69 (30.0)	35 (24.4)	34 (39.0)	0.019*
	Video instruction	26 (11.3)	18 (12.5)	8 (9.2)	0.430
	E-mail update	42 (18.2)	24 (16.7)	18 (20.6)	0.457
	Social network	14 (6.0)	8 (5.5)	6 (6.9)	0.688
Exercise guide for pain conditions	Mobile app	105 (45.6)	65 (45.45)	40 (45.9)	0.938
	Pocket card	74 (32.1)	42 (29.3)	32 (36.7)	0.243
	Video instruction	81 (35.2)	54 (37.7)	27 (31.03)	0.300
	E-mail update	61 (26.5)	37 (25.8)	24 (27.5)	0.775
	Social network	33 (14.3)	21 (14.6)	12 (13.7)	0.851
Mindfulness/meditation	Mobile app	108 (46.9)	64 (44.7)	44 (50.7)	0.391
	Pocket card	52 (22.6)	26 (18.1)	26 (29.8)	0.039*
	Video instruction	96 (41.7)	59 (41.2)	37 (42.5)	0.849
	E-mail update	50 (21.7)	32 (22.3)	18 (20.6)	0.763
	Social network	43 (18.6)	30 (13.9)	13 (14.9)	0.841
Motivational interviewing	Mobile app	81 (35.2)	47 (32.8)	34 (39.08)	0.338
	Pocket card	49 (21.3)	24 (16.7)	25 (28.7)	0.031*
	Video instruction	98 (42.6)	54 (37.7)	44 (50.5)	0.056
	E-mail update	51 (22.1)	30 (20.98)	21 (24.14)	0.576
	Social network	41 (17.8)	23 (16.0)	18 (20.6)	0.371
Nonpharmacological treatments	Mobile app	111 (48.2)	65 (45.4)	46 (52.8)	0.274
	Pocket card	73 (31.7)	39 (27.2)	34 (39.0)	0.062
	Video instruction	86 (26.0)	54 (37.7)	32 (36.7)	0.881
	E-mail update	68 (29.5)	42 (29.3)	26 (29.8)	0.933
	Social network	44 (19.1)	27 (18.8)	17 (19.4)	0.901
Local pain resources/treatment services	Mobile app	90 (39.1)	55 (38.4)	35 (40.2)	0.789
	Pocket card	55 (23.9)	38 (26.5)	17 (19.5)	0.225
	Video instruction	36 (15.6)	25 (17.4)	11 (12.6)	0.327
	E-mail update	78 (33.9)	45 (31.4)	33 (37.9)	0.315
	Social network	44 (19.1)	28 (19.5)	16 (18.3)	0.824

*P < 0.05 was considered statistically significant.

## Discussion

In our survey of Canadian HCPs, we found self-reported CPD learning needs related to pain distributed across all IASP competency domains. The results supported how the IASP endorsed pain competencies are a relevant framework for beginning to consider CPD needs/interests among postlicensure HCPs. Respondents reported that more than half of all patients encountered in an average week presented with pain; this finding emphasizes the importance of knowledgeable HCPs to ensure safe and effective pain management. Most reported moderate competency in assessing acute pain and routine use of unidimensional pain assessment tools. Competency in appraising chronic pain was lower, albeit not significantly lower, than for acute pain. Multidimensional pain appraisal tools were not routinely incorporated into practice. Recently completed pain learning activities and future learning preferences were most often informal (e.g., reading journal articles, attending conferences) and work-based (e.g., hospital rounds) in nature.

As hypothesized, HCPs working in academic settings had greater access to resources such as pain specialists/teams and higher self-rated acute pain competency in comparison to peers working in nonacademic settings. The knowledge and skill to assess both acute and chronic pain are a critical component of accurate pain classification and a core IASP-endorsed competency. Our results demonstrate room for improving HCP skill and confidence, particularly in chronic pain assessment, which is important in the context of an aging population and growing prevalence of complex pain conditions.^23^ Primary care providers report chronic pain as one of the most challenging conditions to treat, yet it is given limited attention in any HCP curriculum in relation to the societal burden it imposes.^24^ Up to 25% of Canadians have chronic pain, which is associated with functional and social impairment. HCP interest in learning more about multidimensional pain appraisal is relevant for diagnosing chronic pain and understanding its treatment options.

The top identified condition for continuing knowledge development among those caring for adult and older adult populations was neuropathic pain, which has a high impact on quality of life and increasing prevalence in North America.^25^ Compared with other pain issues, neuropathic pain is likely to be rated as more severe.^26^ Symptoms of neuropathic pain such as burning, numbness, and allodynia can be difficult for patients to describe. This may be particularly true of institutionalized older adults, who often experience self-report incapacities.^27^ Taken together, these issues may contribute to problems diagnosing neuropathic pain outside of specialty settings. Moreover, the modest to moderate benefit of conventional analgesics for neuropathic pain may factor into treatment uncertainties among HCPs and lack of pain relief among patients.^28^ HCP trainees in Canada have limited exposure to patients with persistent chronic pain conditions. This training gap may leave them unclear about appropriate care in practice.^29^

The most frequently identified competency development needs for those caring for adolescents and children/infants were musculoskeletal pain and visceral pain, respectively. Drivers of help-seeking for musculoskeletal pain appear to be severe pain intensity and limited activity; up to 37% of children and adolescents report visiting a clinician for such pain in the past year.^30^ Similarly, abdominal (e.g., visceral) pain is a common problem among children.^31^ Those experiencing chronic abdominal pain report significantly lower quality of life compared to their healthy counterparts and are more frequently absent from school.^32^ There is emerging evidence that children and adolescents who report persistent pain are at increased risk of chronic pain as adults.^30^ These commonly presenting pain conditions represent practical learning needs and a likely opportunity to influence both practice and patient outcomes through targeted CPD.

Years in practice is not necessarily a determinant of competence in pain management.^33^ Instead, HCPs’ capacities to successfully deliver patient-centered pain care are predicated upon foundational training and an environment that supports continuous professional development.^4^ Current IASP recommendations include multidisciplinary and interprofessional learning approaches, whereby shared understandings of pain mechanisms and biopsychosocial concepts contribute to collaborative and comprehensive patient treatment.^34^ The informal approaches reported by respondents in this study (e.g., reading journal articles) may be ineffective in changing or reinforcing one’s capacity to perform a task. Effective professional development experiences are most often interactive, multimodal, and cyclic and incorporate performance feedback.^35^ This aligns with growing scientific evidence that passive implementation strategies are less effective than active ones.^36^ Therefore, it is appropriate for HCPs to reflect on the IASP competency domains to identify priority learning needs and effective modalities for enhanced care delivery.^37^

Improving HCP skill and confidence in assessment and managing pain is important for equitable health outcomes. In Ontario, Canada’s most populated province, approximately 55% of all hospital beds are in large (nonacademic) community hospitals, defined as having more than 100 beds. These community hospitals provide medical and surgical care to >65% of all patients in the province annually.^38^ In contrast, the majority (91.3%) of multidisciplinary specialist pain clinics that provide access to multimodal care for people living with chronic pain are concentrated in large urban cities, with almost two-thirds (64.9%) being university affiliated.^39^ In our study, the lower reported HPC access to acute and chronic pain specialists in nonacademic work settings may unintentionally contribute to either failure or delay in the implementation of evidence-based pain management. This issue may exacerbate known inequities in pain treatment and outcomes,^11^ especially for those in rural settings who may experience additional barriers to accessing health care.^40^

The Canadian Pain Task Force was established in 2019 to better understand and address the needs of people living with chronic pain.^1^ The Canadian Pain Task Force strongly recommends that health professionals continually develop the competencies they require to successfully provide effective pain care. The wide range of learning needs identified in our study point to the importance of health system investment and organizational leadership facilitation of CPD in pain. When CPD is driven solely by professional registration requirements, it can lead to a dissociation from lifelong learning.^41^ For many HCPs, personal motivation, real-world patient care needs, and preference for workplace learning are important drivers of CPD.^42^ Strong enabling leadership and a culture of inquiry are important for translating evidence into clinical practice.^43^ This may be particularly important in nonacademic and rural/remote clinical settings, which may lack a range of resources (e.g., pain specialist/teams, education staff, researchers) that are more readily available in larger urban settings. HCPs working in rural settings may experience barriers to leaving their region to attend CPD opportunities, thus pointing to the importance of organizational investment in workplace learning.

The potential of digital CPD modalities to improve HCP access to learning opportunities has been recently accelerated by the COVID-19 pandemic. Since 2020, in-person meeting restrictions and pandemic-related travel embargoes have limited access to conventional CPD opportunities such as local, national, and international conferences and hospital rounds/case discussions. HCPs have responded positively to digital conferences and e-learning courses because they can be less costly and taken at time suited to their schedule.^44^ These changes may align with advances in clinical learning sciences, which now emphasize how flexible learning opportunities can increase participation.^45^ Application of knowledge and feedback mechanisms can be incorporated in digital learning so that participants can reflect upon their developing knowledge and skill.^33^ Digital formats can also reinforce knowledge uptake through recurrent access to learning content. Both academic and nonacademic health care institutions should consider implementation of digital CPD opportunities in pain management.

Strengths of the study include the interprofessional survey design, high survey return rate, and the inclusion of diverse groups of HCPs. Aspects of our survey methods leave open the possibility that our findings do not represent the larger population of Canadian HCPs; participants may overrepresent nurses, those with greater years of professional experience, and HCPs working in academic hospital settings. The eight-week response period may have biased results to early responders and the web-only administration format may exclude those who do not regularly check e-mail or are less comfortable with online interactions. Tailoring items and response frames to each HCP group and/or clinical population could have enhanced relevance. Although we evaluated the face and content validity of the survey, some items may not have been understood equally. As with all surveys, self-reported practices may not align with actual practices. Finally, our survey predated the COVID-19 pandemic, which means that learning needs may have evolved.

## Conclusion

In this cross-sectional survey of Canadian postlicensure HCPs, respondents frequently encountered patients experiencing pain. They identified their learning needs to improve the management of complex and persistent pain conditions. IASP-endorsed interprofessional pain competencies offer a productive framework for assessing HCP learning needs and identifying domains for targeted knowledge development. Greater access to CPD and pain experts using a number of learning modalities is warranted to facilitate evidence-based pain management and optimal patient outcomes.

## Supplementary Material

Supplemental Material

Supplemental Material

## Data Availability

The data sets used and/or analyzed during the current study are available from the corresponding author on reasonable request.
