# Supplementary material for: Continuing professional development needs in pain management for Canadian health care professionals: A cross sectional survey
Source: Can J Pain. 2023 Jan 23;7(1):2150156. doi: 10.1080/24740527.2022.2150156 (PMC9872952; doi:10.1080/24740527.2022.2150156)
Supplement: Supplemental Material [file UCJP_A_2150156_SM8843.pdf]

Background Information

1. Are you a Regulated Health Professional?

☐ No ☐ Registered Nurse ☐ Nurse Practitioner ☐ Pharmacist
☐ Physician ☐ Occupational Therapist ☐ Physician Assistant ☐ Physiotherapist
☐ Dentist ☐ Dental Hygienist ☐ Speech Language ☐ Dietitian
☐ Other (Specify): ____

2. What is your highest level of education completed?

☐ Diploma/College ☐ Baccalaureate ☐ Master's Degree
☐ Doctorate/PhD ☐ Post-Doctorate
☐ Other (Specify): ____

3. How many years have you been practicing in your profession since qualifying?

☐ 0-5 years ☐ 6-10 years ☐ 11-20 years
☐ 21-30 years ☐ > 30 years

4. In what type of clinical setting do you practice? *Check all that apply*

☐ University-affiliated hospital ☐ Community hospital ☐ Urgent care clinic
☐ Private office/clinic ☐ Complex/continuing care ☐ Rehabilitation
☐ Hospice ☐ Pharmacy
☐ Other (specify): ___________________________________

5. What patient populations and conditions do you encounter in your practice? *Check all that apply*

☐ **Infants/children**
☐ **Adolescents**
☐ **Adults**
☐ **Older Adults**

☐ Palliative conditions ☐ Critical illness ☐ Mental illness
☐ Endocrine disorders ☐ Cardiovascular disease ☐ Musculoskeletal problems
☐ Organ failure/transplant ☐ Cancer ☐ Neurological disease
☐ Traumatic injury ☐ Oral/craniofacial disease ☐ Respiratory disease
☐ Infectious disease ☐ Cognitive impairment ☐ Substance use disorder
☐ Other (Specify): ____

6. How many patients do you care for in an average week?

☐ < 50 ☐ 51-100 ☐ 101-150 ☐ >150

7. How many of these patients might present with pain in an average week?

☐ 0 -10% ☐ 11-20% ☐ 21-30% ☐31-40%
☐ 41-50%% ☐ >50%

8. I have access to the following resources in my practice setting:

Acute Pain Specialist/Team	☐ Yes	☐ No	☐ Uncertain
Chronic Pain Specialist/Team	☐ Yes	☐ No	☐ Uncertain

☐ Other (Specify): ____

9. On a scale of 0 to 10, how competent do you feel in carrying out an <u>acute pain</u> assessment in your clinical setting?

Not competent — Highly competent

0 1 2 3 4 5 6 7 8 9 10

10. On a scale of 0 to 10, how competent do you feel in carrying out a <u>chronic pain</u> assessment in your clinical setting?

Not competent — Highly competent

0 1 2 3 4 5 6 7 8 9 10

Pain Management Resources

11. Which tools do you use to assess pain in your clinical setting? *Check all that apply*

☐ None ☐ Numeric Rating Scale ☐ Visual Analog Scale ☐ Verbal Rating Scale
☐ Behavioral Pain Scale ☐ Critical-Care Pain Observational Tool ☐ PQRST Method
☐ Faces Pain Scale ☐ Faces, Legs, Activity, Cry, Consolability Scale ☐ Premature Infant Pain Profile-Revised
☐ Brief Pain Inventory ☐ McGill Questionnaire ☐ Pain Disability Index
☐ Other (Specify): ____

12. Which resources do you use to make treatment choices for pain? *Check all that apply*

☐ None ☐ Clinical order sets ☐ Pain protocol/policy
☐ Patient pain goal ☐ Physiological testing ☐ Published guideline
☐ Patient self-report ☐ Quantitative sensory testing ☐ Urine Drug screen
☐ Professional consultation ☐ Online resources
☐ Other (Specify): ____

13. What are the potential barriers to the optimal treatment of pain in your clinical practice setting? *Check all that apply*

☐ None
☐ Potential side effects of analgesia
☐ Under-treatment of pain
☐ Ineffective clinical order set
☐ Lack of time
☐ Institutional pain leadership
☐ Lack of guideline awareness
☐ Potential drug interactions
☐ Over-treatment of pain
☐ Lack of treatment options
☐ Clinician pain knowledge
☐ Low importance of pain
☐ Competing priorities
☐ Fear of opioid use disorder
☐ Patient/family issues
☐ Costs to patients
☐ Short-staffing
☐ High patient acuity
☐ Other (Specify): ____

Future professional development

14. Please identify the educational topics related to the multidimensional nature of pain that could help you improve your abilities to manage pain. *Check all that apply*

☐ None
☐ Pain terminology/classification
☐ Ethical standards
☐ Pain epidemiology
☐ Pain physiology
☐ Legal issues
☐ Pain theory
☐ Consequences of pain
☐ Other (Specify): ____

15. Please identify the educational topics related to the assessment and measurement of pain that could help you improve your abilities to manage pain. *Check all that apply*

☐ None
☐ Laboratory/imaging investigations
☐ Interprofessional collaboration
☐ Pain assessment tools/scales
☐ History and physical exam
☐ Tool reliability/validity
☐ Other (Specify): ____

16. Please identify the educational topics related to the management of pain that could help you improve your abilities to manage pain. *Check all that apply*

☐ None
☐ Patient/family beliefs
☐ Pharmacological methods
☐ Goals of pain management
☐ Access to treatment issues
☐ Psychological/Cognitive methods
☐ Pain management planning
☐ Psychological issues
☐ Substance use disorder
☐ Other (Specify): ____

17. Please identify the educational topics related to special populations/clinical conditions that could help you improve your abilities to manage pain. *Check all that apply*

☐ **Infants/children**
☐ **Adolescents**
☐ **Adults**
☐ **Older adults**

☐ None
☐ Cancer pain
☐ Substance use disorders
☐ Visceral pain
☐ Mental health
☐ Pregnancy/breast feeding
☐ Acute/time-limited pain
☐ Headache

☐ Neuropathic pain
☐ Musculoskeletal pain
☐ Orofacial pain
☐ Chronic pain

☐ Other (Specify): ____

18. Please identify tools, if any, that could help improve your abilities to manage pain.

	Pocket Card	Mobile App	Email Updates	Video Instruction	Social Network	Other format (specify)
Acute pain assessment	☐	☐	☐	☐	☐	
Analgesic dosing guide	☐	☐	☐	☐	☐	
Chronic pain assessment	☐	☐	☐	☐	☐	
Drug interactions	☐	☐	☐	☐	☐	
Exercise guide for pain-related conditions	☐	☐	☐	☐	☐	
Guideline implementation tools	☐	☐	☐	☐	☐	
Mindfulness/ meditation	☐	☐	☐	☐	☐	
Motivational interviewing/ solution focused coaching	☐	☐	☐	☐	☐	
Non-pharmacological modalities	☐	☐	☐	☐	☐	
Local/ regional resources or services	☐	☐	☐	☐	☐	
Other (Specify):	☐	☐	☐	☐	☐	

19. In the last 12 months, how have you stayed abreast of new pain science, protocols or evidence-based recommendations related to pain assessment and management in your practice? *Check all that apply*

☐ None
☐ National conferences
☐ Local conferences
☐ Hospital rounds
☐ Journal club
☐ Online independent learning
☐ Webcasts (live)
☐ Reading journal articles
☐ Reading textbooks
☐ Webcasts (recorded)
☐ Email
☐ Simulation
☐ Formal courses
☐ Social media (e.g., Twitter)
☐ UTCSP website
☐ Other (Specify): ____

20. What is your preferred format/method of learning? *Check all that apply*

☐ None
☐ National conferences
☐ Local conferences
☐ Hospital rounds
☐ Journal club
☐ Online independent learning
☐ Webcasts (live)
☐ Reading journal articles
☐ Reading textbooks
☐ Webcasts (recorded)
☐ Email
☐ Simulation
☐ Formal courses
☐ Social media (e.g., Twitter)
☐ UTCSP website
☐ Other (Specify): ____

Thank you for taking part in the survey. Results will be available through the UTCSP website.
